# Modelling heterogeneity in the classification process in multi‐species distribution models can improve predictive performance

**DOI:** 10.1002/ece3.11092

**Published:** 2024-03-07

**Authors:** Kwaku Peprah Adjei, Anders Gravbrøt Finstad, Wouter Koch, Robert Brian O'Hara

**Affiliations:** ^1^ Department of Mathematical Sciences Norwegian University of Science and Technology Trondheim Norway; ^2^ Center for Biodiversity Dynamics Norwegian University of Science and Technology Trondheim Norway; ^3^ Norwegian Institute for Nature Research Trondheim Norway; ^4^ Department of Natural History Norwegian University of Science and Technology Trondheim Norway; ^5^ Norwegian Biodiversity Information Centre Trondheim Norway

**Keywords:** Bayesian models, citizen science, false positives, machine learning, misclassification, multi‐species distribution models

## Abstract

Species distribution models and maps from large‐scale biodiversity data are necessary for conservation management. One current issue is that biodiversity data are prone to taxonomic misclassifications. Methods to account for these misclassifications in multi‐species distribution models have assumed that the classification probabilities are constant throughout the study. In reality, classification probabilities are likely to vary with several covariates. Failure to account for such heterogeneity can lead to biased prediction of species distributions. Here, we present a general multi‐species distribution model that accounts for heterogeneity in the classification process. The proposed model assumes a multinomial generalised linear model for the classification confusion matrix. We compare the performance of the heterogeneous classification model to that of the homogeneous classification model by assessing how well they estimate the parameters in the model and their predictive performance on hold‐out samples. We applied the model to gull data from Norway, Denmark and Finland, obtained from the Global Biodiversity Information Facility. Our simulation study showed that accounting for heterogeneity in the classification process increased the precision of true species' identity predictions by 30% and accuracy and recall by 6%. Since all the models in this study accounted for misclassification of some sort, there was no significant effect of accounting for heterogeneity in the classification process on the inference about the ecological process. Applying the model framework to the gull dataset did not improve the predictive performance between the homogeneous and heterogeneous models (with parametric distributions) due to the smaller misclassified sample sizes. However, when machine learning predictive scores were used as weights to inform the species distribution models about the classification process, the precision increased by 70%. We recommend multiple multinomial regression to be used to model the variation in the classification process when the data contains relatively larger misclassified samples. Machine learning prediction scores should be used when the data contains relatively smaller misclassified samples.

## Graphical abstract

This study presents a multi‐species distribution model that accounts for heterogeneity in the classification process. We found out that accounting for the heterogeneity improves the predictive performance of the species distribution models.

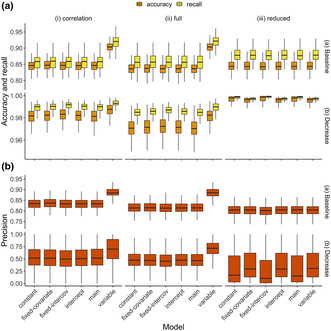

## 1 INTRODUCTION

Species distribution models are essential ecology and conservation management tools that predict how natural and human factors affect biodiversity (Elith & Leathwick, 2009; Vermeiren et al., 2020). With increasing biodiversity data from multi‐species surveys available to scientists, multi‐species distribution models (hereafter mSDMs) and joint species distribution models (jSDMs) have become widely used in analysing these data to identify the important variables that drive species co‐occurrences and predict the distribution of species in a community (Hui et al., 2015; Ovaskainen & Soininen, 2011; Pollock et al., 2014). These mSDMs model data at the community level by identifying how individual taxa respond to environmental variables (Ovaskainen & Soininen, 2011). The jSDMs also quantify the residual correlation between taxa after the explanatory variables have been accounted for (Caradima et al., 2019; Pollock et al., 2014).

However, the biodiversity data obtained from these surveys can be subject to observation errors, and misclassification is a common source of this error. The misclassification may arise from imperfect classifiers (Spiers et al., 2022; Wright et al., 2020), observer error and many other sources. Species misclassification in multi‐species surveys often involves reporting one species as another, resulting in false positives (where the species whose identity has been reported is actually absent; Miller et al., 2011; Royle & Link, 2006) and false negatives (where the species whose identity was misclassified is present but reported as absent; MacKenzie et al., 2002). In this study, we use the term true states to describe the correct or actual observation identity we are interested in modelling. Although it is not always possible to know if individuals are correctly classified or not, it would be a great advantage if the observations were correctly classified (for example, through predictions from fitted species distribution that account for misclassification) rather than discarded once they were identified as false positives. False negatives and positives are mostly accounted for in occupancy models by jointly modelling them in the observation model (Kéry & Royle, 2020; Miller et al., 2011; Royle & Link, 2006). Failure to account for or correct these errors leads to biases in inferences about state variables such as occupancy probabilities, covariate effects and relative activity (Clare et al., 2021; Ferguson et al., 2015; Miller et al., 2015; Royle & Link, 2006; Wright et al., 2020), leading to an impairment in decision making (Hoekman, 2021).

The methods to deal with misclassification from biodiversity data can be grouped into data review methods and model‐based methods (Clare et al., 2021). Data review methods require complete and proper data collection and processing methods. This process can be very demanding as it is challenging to control for misclassification. This makes the model‐based methods more popular when working with large‐scale datasets from large‐scale biodiversity data vendors like the Global Biodiversity Information Facility (GBIF hereafter; GBIF.Org, 2022). Model‐based methods estimate classification probabilities jointly with the true state variables of interest. Model‐based methods attempting to account for misclassification in multi‐species occupancy models currently include modelling misclassification with detection heterogeneity (Clement et al., 2022; Ferguson et al., 2015; Louvrier et al., 2018), integrating multiple observers records with other methods such as distance sampling and N‐Mixture models (Hoekman, 2021), supervised methods with extra information from observation confirmation or verification (Ferguson et al., 2015; Guillera‐Arroita et al., 2017), site confirmation (Clare et al., 2021) and other calibrated methods. These methods need extra data from the verification process, which helps in estimating the misclassification probabilities in a semi‐supervised setting (Spiers et al., 2022) and makes the parameters in the model identifiable (Guillera‐Arroita et al., 2017). The above‐mentioned studies have either used verified data collected on the site level (where the occupancy state of a species is known at a site and not at the individual sample level; Chambert, Waddle, et al., 2018), on aggregated individual sample level using a multinomial model with site‐covariates (Wright et al., 2020) or on individual sample‐level validation data which helps in modelling non‐species identities (morphospecies) to species identities (Spiers et al., 2022). It is also worth stating that some studies have explored accounting for misclassification in abundance (Conn et al., 2013), capture–recapture (Augustine et al., 2020) and mixture (Guilbault et al., 2021) models.

Furthermore, these previous studies assumed that the misclassification probabilities are homogeneous (constant) across the study. In reality, the classification probabilities may vary with environmental covariates (such as field conditions; Conn et al., 2013) or observer experience (especially when ascertaining how well each observer classifies a report in citizen science projects will be informative; Arazy & Malkinson, 2021; Johnston et al., 2022), distance from a transect when using transect data (Conn et al., 2013), picture quality, etc. An attempt at modelling the heterogeneity in the classification process is to assume homogeneous classification probabilities and add the classification covariates to the ecological model. However, this approach may not solve the heterogeneity problem in the classification process since the estimates of the ecological process parameters only serve as informed priors to the classification process (Spiers et al., 2022).

A more correct approach to model this heterogeneity is adding the covariate effect to the observation process. Some studies on dynamic false positive single‐species occupancy models have modelled temporal changes in false positives using year as a covariate (Kéry & Royle, 2020; Miller et al., 2013; Sutherland et al., 2013), showing the possibility to model misclassification trends over time. Our study attempts to model variation in classification probabilities in mSDMs by modelling the probability of classifying an individual with a multinomial generalised linear model as a function of covariates. To our knowledge, no previous work has been done on this. Failure to account for the heterogeneity in the misclassification probabilities can lead to biased estimates in the process model (such as species abundance, richness and occupancy probabilities) and reduce the model's predictive performance (Chambert et al., 2015; Spiers et al., 2022; Wright et al., 2020).

Fitting a complex model with many parameters can result in an overfitted model. An overfitted model captures the pattern and noise in the training data but performs poorly on validation or test data (Montesinos López et al., 2022). The ecological process and observation model covariates can sometimes be highly correlated. These correlated covariates can inflate standard errors (reduce the precision) of the estimated parameters (Caradima et al., 2019; Roberts et al., 2017; Yu et al., 2015). To avoid overfitting the model, there is a need to perform variable selection and select the variables that are related to the state variable of interest (Fox et al., 2017; Murtaugh, 2009; O'Hara & Sillanpää, 2009).

Moreover, recent efforts to correctly classify observations from biodiversity surveys have relied on machine learning (hereafter ML) algorithms (Borowiec et al., 2022; Keshavan et al., 2019; Koch et al., 2022; Lotfian et al., 2021; Saoud et al., 2020; Suzuki‐Ohno et al., 2022; Willi et al., 2019). These ML algorithms use sounds and/or images of observations to predict the true identity of the individual observations, and they can be trained to mimic expert verification of observations (Keshavan et al., 2019; Langenkämper et al., 2019; Ponti & Seredko, 2022). These ML algorithms use a prediction score (a value that shows the weight of predicting the observations as something else) to predict the possible list of the true identities of the individual reported observation. These prediction scores and a list of possible true identities provide information about the classification process of each observation. They can be used to model heterogeneity in the classification process. This study is the first to model the heterogeneity in the classification process by using the prediction scores to weigh the distribution of the reported observations and predict the distribution of the actual observation identities.

Here, we present a joint model that simultaneously models the true state variables of interest (relative abundance) and the heterogeneity in the classification process. Our model set‐up extends the work done by Wright et al. (2020) and Spiers et al. (2022) by (a) allowing the classification probabilities to vary with covariates, (b) using ML prediction scores as weights to account for heterogeneity in the classification process and (c) performing variable selection on the classification process covariate to check for potential mSDM overfit. Studies have already been done on comparing models that account for a ‘homogeneous’ classification process to those that do not account for misclassification (Chambert et al., 2015; Spiers et al., 2022; Wright et al., 2020). Therefore, we compare the classification performance of our model with models that assume a homogeneous classification probability done by Wright et al. (2020) and Spiers et al. (2022) through simulation studies and not to models that do not account for misclassification. We parameterise our model with citizen science data on gulls in Norway, Finland and Denmark from iNaturalist (Matheson, 2014) downloaded from the GBIF (GBIF.Org, 2022).

## 2 METHODOLOGY

### 2.1 Model framework

The proposed framework starts by assuming we have individuals who are observed and classified into a state, known as the ‘reported’ or ‘classified’ state (there may be one of many at a location, but each individual is classified with a probability). We use ‘state(s)’ in this work to refer to taxon identity as well as any other identification category or morpho‐states, that is individuals cannot be identified to their taxonomic states and are grouped based on their morphology (Spiers et al., 2022). This state can be on any taxonomic level. We further assume that these individuals are verifiable (and we have information on the verification process) and that the verified state approximates the true state identity (that is, we assume that the verified information is free from misclassification). We describe an observation model for the individuals in Section 2.1.1 and define another model for the ecological process in Section 2.1.2.

#### 2.1.1 Defining the observation model

The observation model in mSDMs usually accounts for observation errors such as imperfect detection, uneven sampling effort, misclassification and many others. In this study, we account for only misclassification in the observation model of our mSDMs. Therefore, we use the term observation and classification process interchangeably.

To describe the observation model, we assume that observations are classified individually, irrespective of the data collection protocol. Each individual observed can be classified into k=1,2,…,K states (where *K* is the number of unique reported states identities of interest), and every reported information can be seen as a draw from the *K* reported states under consideration with a given probability. As mentioned above, these states could be on any taxonomic level or include any unidentified group. For example, one could have four true states: common, herring, Audouin's and Sooty gull. These species can be reported in three states: large white‐headed gulls, large black‐headed gulls and others. It is worth mentioning here that the reported states do not necessarily include the individual species. An example of the classification probability (confusion matrix) is shown in Table 1.

**TABLE 1:** Example of confusion matrix that applies to our model. Individual observations (referred to as reported states) are verified as the true states.

True states	Reported states		
	Large white‐headed gulls	Large black‐headed gulls	Other gulls
Common gull	0.8	0.1	0.1
Herring gull	0.9	0	0.1
Audouin's gull	0	0.9	0.1
Sooty gull	0	1	0

Let $\Omega_{\mathrm{jk}}$ be the probability that an individual true state $j\in \left\{1,,,\ldots ,,,J\right\}$ (where *J* is the number of unique true states identities of interest) is classified as state $k\in \left\{1,,,\ldots ,,,K\right\}$. The probabilities across all the possible *k* states sum to 1. In studies with homogeneous classification probabilities, the confusion matrix for the classification can be expressed as:
(1)

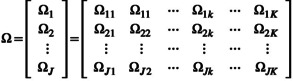

where the rows correspond to the true state *j* and the columns correspond to the reported states *k*.

We model the heterogeneity in the classification probabilities by fitting a multinomial generalised linear model (Fahrmeir et al., 2013) to each of the rows of Ω defined in Equation (1). We refer to this approach as the multiple multinomial generalised linear model (MMGLM, hereafter). For each individual *s* observed at a location (which can be fixed as in transects or breeding‐bird survey fixed points or random), we define the linear predictor of the MMGLM as:

(2) $$\begin{array}{c} \zeta_{\mathrm{jks}}=\omega_{0\mathrm{jk}}+\sum_{p=1}^{n}z_{\mathrm{ps}}\times \omega_{\mathrm{pjk}}\\ =\left[\begin{array}{cccc} \omega_{011} & \omega_{012} & \cdots & \omega_{01K}\\ \omega_{021} & \omega_{022} & \cdots & \omega_{02K}\\ \vdots & \vdots & \cdots & \vdots \\ \omega_{0J1} & \omega_{0J2} & \cdots & \omega_{0\mathrm{JK}} \end{array}\right]+\ldots +\left[\begin{array}{cccc} \omega_{n11} & \omega_{n12} & \cdots & \omega_{n1K}\\ \omega_{n21} & \omega_{n22} & \cdots & \omega_{n2K}\\ \vdots & \vdots & \cdots & \vdots \\ \omega_{\mathrm{nJ}1} & \omega_{\mathrm{nJ}2} & \cdots & \omega_{\mathrm{nJK}} \end{array}\right]\times z_{\mathrm{ns}} \end{array}$$

where $\omega_{0\mathrm{jk}}$ is the intercept of the *j*th true state and *k*th reported state and $\omega_{\mathrm{pjk}}$ is the *j*th true state and *k*th reported state effect of covariate $z_{\mathrm{ps}}$ for individual *s* with the covariate index $p\in \left\{1,2,\ldots n\right\}$, with *n* being the number of covariates that drives the observation process. Using Equation (2) as the definition for the linear predictor, our estimates of the parameters $\omega_{0\mathrm{jk}}$ and $\omega_{\mathrm{pjk}}$ are identifiable with reference to one reported state. That is, for each observed individual *s* and true state identity *j*, the classification probabilities ($\Omega_{\mathrm{jks}}$) for each reported state k=1,2,…,K−1 with reference to state *K* is modelled as the logarithm of the ratio of linear predictors defined in Equation (2):
(3)

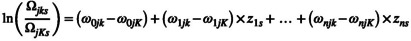

with the same definition of model parameters in Equation (2). The derivation of Equation (3) from Equation (2) is shown in Appendix S1.

This general framework has *J* × (*K*−1) × (*n*−1) parameters to be estimated, where *J* is the number of true states, *K* is the number of reported states, and *n* is the number of covariates in the observation model. Estimating these parameters can be very computationally expensive as the number of true states, reported states and covariates increase, requiring significant numbers of misclassified individuals to estimate them. Therefore, we explored simplified forms of the generalised model in Equation (3).

A simplified case of Equation (3) assumes that the covariate $z_{\mathrm{ps}}$ only affects the probability of correctly classifying individuals. For example, when we want to model the heterogeneity in the classification probabilities through the probability of correctly classifying the species. In this instance, $\omega_{\mathrm{pjk}}=0$ for j≠k for covariate *p*, and these parameters are not estimated (This is our study scenario ‘fixed covariate’ in Table 2). This simplification reduces the number of parameters estimated for the observation process by $n\times \left(K-J-1\right)$, where *J* is the number of true states, *K* is the number of reported states, and *n* is the number of observation model covariates. A further simplification would also be to assume that, on average, all the true states have the same probability of being correctly classified; that is, $\omega_{0\mathrm{jk}}$ is the same for all j=k (This is our study scenario ‘fixed‐intercov’ in Table 2). As such, the covariate effect $\omega_{1\mathrm{jk}}$ for all j=k captures the classification process heterogeneity. The latter further reduces the number of parameters estimated by *J*–1. This last simplification is useful, especially when individuals from different states are very similar, and one would expect their average classification probabilities to be the same.

Then, given that an individual *s* was sampled, the reported state of that individual is a draw from *K* states with probability $\Omega_{j.s}$:

(4) $$Y_{s}|V_{s}=j∼\text{Categorical}\left(\Omega_{j\cdot s}\right)$$

where $Y_{s}$ is the reported observation identity and $V_{s}$ is the true state identity obtained from the verification process for individual *s*.

#### 2.1.2 Ecological process model

We now define an ecological process model for the true state distribution. Although we have assumed that the reported observations are classified on individual levels, the definition of the process model can either be on an individual sample level (that is, individual true state information is present at each site, such that data obtained from a species list) or an aggregate sample level (for example, counts of all individuals across all true states at a location).

We specify a relative abundance model (specifically a multinomial logit model) for each true state's ecological process. Our objective is to show how to model heterogeneity in the classification process and not to make inferences about the true state's abundance, so we chose a model that was easier to fit and understand to describe the ecological process.

Let $\lambda_{\mathrm{js}}$ be the average number of individuals in true state j=1,2,…,J for individual *s*, which describes the abundance of the individuals over the study region *D*. This intensity can either be modelled as an inhomogeneous process, which assumes that the data are dependent on the environment covariate, or as a log‐Cox Gaussian Point process, where we assume a spatial dependency in the observed data (Renner et al., 2015). Here, the mean intensity is modelled using the inhomogeneous process and defined as:

(5) $$\ln\left(\lambda_{\mathrm{js}}\right)=\beta_{0j}+\sum_{q=1}^{n_{e}}x_{\mathrm{qs}}\times \beta_{\mathrm{qj}},$$

where $\beta_{0j}$ is the intercept of state j, $\beta_{\mathrm{qj}}$ is the effect of covariate with index $q\in \left\{1,2,\ldots ,n_{e}\right\}$ on the intensity of true state j, $x_{\mathrm{qs}}$ is the *q*th covariate that affects the observation individual *s* and $n_{e}$ is the number of covariates in the ecological process model. Note that we assume there are no species interactions or residual correlation in our relative abundance model, and this could have been added as a random effect in the true state intensity definition (Equation (5)).

Let $p_{\mathrm{js}}$ be the relative proportion (probability) that an individual *s* belongs to true state *j*. We estimate this probability from the mean intensities as follows:

(6) $$p_{\mathrm{js}}=\frac{\lambda_{\mathrm{js}}}{\sum_{j}^{J}\lambda_{\mathrm{js}}},$$

where $\lambda_{\mathrm{js}}$ is defined in Equation (5).

The true state of each individual observation *s* is a realisation from a categorical distribution with probability $p_{\mathrm{js}}$. This distribution assumption indicates that we assume a single true state for every individual. When aggregate sample level data is available instead of individual sample data, then the total number of individuals in each true state follows a Poisson distribution with parameter λ (as defined in Equation 5), and within that, the number of each recorded state follows a multinomial distribution with probabilities Ω (as described in Section 2.1.1). This implies that there can be multiple individuals at each site, except that each of these individuals shares the same site‐specific covariates.

In summary, the hierarchical framework of the proposed mSDMs is as follows:

(7) $$\begin{array}{l} \;\ln\left(\lambda_{\mathrm{js}}\right)=\beta_{0j}+\sum_{q=1}^{n_{e}}x_{\mathrm{qs}}\beta_{\mathrm{qj}};\\ \;p_{\mathrm{js}}=\frac{\lambda_{\mathrm{js}}}{\sum_{j}\lambda_{\mathrm{js}}};\\ \;V_{s}∼\text{Categorical}\left(p_{.s}\right);\\ \ln\left(\frac{\Omega_{\mathrm{jks}}}{\Omega_{\mathrm{jKs}}}\right)=\left(\omega_{0\mathrm{jk}}-\omega_{0\mathrm{jK}}\right)+\left(\omega_{1\mathrm{jk}}-\omega_{1\mathrm{jK}}\right)\times z_{1s}+\ldots +\left(\omega_{\mathrm{njk}}-\omega_{\mathrm{njK}}\right)\times z_{\mathrm{ns}};\\ \;Y_{s}|V_{s}∼\text{Categorical}\left(\Omega_{V_{s},\cdot }\right), \end{array}$$

where the definition of parameters is inherited from the models defined in Equations ((3), (4), (5), (6)).

This model specification for the ecological process used here is similar to the occupancy dynamics and encounter rate model used by Spiers et al. (2022) by eliminating the occupancy sub‐model in the ecological process model; and similar to the model used by Wright et al. (2020) by assuming Poisson counts with intensity $\lambda_{\text{is}}$ (refer to Table 3 for the link between our model framework and that of Spiers et al. (2022) and Wright et al. (2020)).

#### 2.1.3 Variable selection

In this study, we performed Bayesian variable selection, specifically the spike and slab prior to the classification process covariates (for review of Bayesian variable selection see O'Hara & Sillanpää, 2009). For each of the classification process covariates, we re‐define the linear predictor in Equation (2) as:
(8)

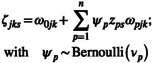

where $\psi_{p}$ is the indicator that variable *p* is selected with the expected probability $\nu_{p}$. The variable selection indicator specified in Equation (8) jointly selects the variables affecting all the true states in the model but can also be state‐specific (Ovaskainen & Abrego, 2020). Probabilities $\nu_{j}$ closer to 1 indicate that the variable contributes much to the model and should be selected, and those closer to 0 indicate that the variable contributes less and can be discarded.

### 2.2 Modelling heterogeneity using ML prediction scores

Some studies give weight to the true identities of the reported observations or state, for example, because they use machine learning to classify the observation. These prediction scores (such as *F*_1_ score, mean square error and logarithmic loss) are not classification probabilities but are values that indicate how well the ML algorithm classifies data in the test sample. In comparison to the model presented in Section 2.1.1, 2.1.2, 2.1.3, the information available here are the categories of the observed or reported individuals and prediction scores, and we are interested in predicting the true identity of the reported individuals. We can use this information to model the heterogeneity in the classification process and predict the true state identity of individuals as follows:

(9) $$\begin{array}{c} \;Y_{s}∼\text{Categorical}\left(\frac{\lambda_{\mathrm{ks}}}{\sum_{k}\lambda_{\mathrm{ks}}}\right)\\ \;V_{s}|Y_{s}∼\text{Categorical}\left(p_{\mathrm{jks}}\right);\\ \text{with}\;p_{\mathrm{jks}}=\frac{\lambda_{\mathrm{ks}}w_{\mathrm{kjs}}}{\sum_{k}\lambda_{\mathrm{ks}}w_{\mathrm{kjs}}} \end{array}$$

where $\lambda_{\mathrm{ks}}$ is the intensity of reported state *k* for individual *s* and $w_{\mathrm{kjs}}$ is the predictive score of the *k*th reported state to true state *j* for individual *s*. The intensity of the reported state is modelled as an inhomogeneous process with covariate effects, similar to the intensity of the true state in Equation (5).

It must be noted that this approach is a non‐parametric approach to account for heterogeneity in the classification whereas the MMGLM is a parametric approach. Moreover, modelling the heterogeneity in the classification process using ML prediction scores models the covariate effects on the expected abundance of the reported states and corrects them using the prediction scores as weights to obtain the relative abundance of the true states. However, the MMGLM models the covariate effect on the expected abundance of true states and estimates the heterogeneity in the classification process using a parametric model. The prediction of the true state identity is done by weighing the expected intensity of true states with the estimated classification covariate.

**TABLE 2:** Variations in the MMGLM for the observation model defined by Equation (8) and ecological process model defined by Equation (5) used as our study scenarios, with one covariate used for each model.

Classification probability type	Study scenario	Ecological process model	Observation process model
Heterogeneous	Variable/covariate	$\ln\left(\lambda_{\mathrm{js}}\right)=\beta_{0j}+x_{1s}\beta_{1j}+x_{2s}\beta_{2j}$	$\ln\left(\frac{\Omega_{\mathrm{jks}}}{\Omega_{\mathrm{jKs}}}\right)=\left(\omega_{0\mathrm{jk}}-\omega_{0\mathrm{jK}}\right)+\psi z_{1s}\left(\omega_{1\mathrm{jk}}-\omega_{1\mathrm{jK}}\right)$
	Fixed covariate	$\ln\left(\lambda_{\mathrm{js}}\right)=\beta_{0j}+x_{1s}\beta_{1j}+x_{2s}\beta_{2j}$	$\ln\left(\frac{\Omega_{\mathrm{jks}}}{\Omega_{\mathrm{jKs}}}\right)=\left(\omega_{0\mathrm{jk}}-\omega_{0\mathrm{jK}}\right)+\psi z_{1s}\omega_{1\mathrm{jj}},$
			where $\omega_{1\mathrm{jk}}=0$ for j≠k
	Fixed intercov	$\ln\left(\lambda_{\mathrm{js}}\right)=\beta_{0j}+x_{1s}\beta_{1j}+x_{2s}\beta_{2j}$	$\ln\left(\frac{\Omega_{\mathrm{jks}}}{\Omega_{\mathrm{jKs}}}\right)=\left(\omega_{0\mathrm{jk}}-\omega_{0\mathrm{jK}}\right)+\psi z_{1s}\omega_{1\mathrm{jj}},$
			where $\omega_{1\mathrm{jk}}=0$ for j≠k
			and $\omega_{0\mathrm{jk}}$ is the same for j=k
Homogeneous	Intercept	$\ln\left(\lambda_{\mathrm{js}}\right)=\beta_{0j}+x_{1s}\beta_{1j}+x_{2s}\beta_{2j}$	$\ln\left(\frac{\Omega_{\mathrm{jks}}}{\Omega_{\mathrm{jKs}}}\right)=\omega_{0\mathrm{jk}}$
	Constant	$\ln\left(\lambda_{\mathrm{js}}\right)=\beta_{0j}+x_{1s}\beta_{1j}+x_{2s}\beta_{2j}$	$\Omega_{\mathrm{jk}}∼\text{Dirichlet}\left(\alpha_{\mathrm{jk}}\right),$
			where $\alpha_{\mathrm{jk}}∼\mathrm{Exp}\left(1\right)$
	Main	$\ln\left(\lambda_{\mathrm{js}}\right)=\beta_{0j}+x_{1s}\beta_{1j}+x_{2s}\beta_{2j}+\psi z_{1s}\beta_{3j}$	$\Omega_{\mathrm{jk}}∼\text{Dirichlet}\left(\alpha_{\mathrm{jk}}\right),$
			where $\alpha_{\mathrm{jk}}∼\mathrm{Exp}\left(1\right)$

*Note*: There are three heterogeneous models: covariate, fixed covariate and fixed intercov and three homogeneous models: intercept, constant and main. The definitions of the parameters in this table are described in Sections 2.1.1, 2.1.2, 2.1.3.

### 2.3 Generalisation of model framework

The classification component of the proposed framework generalises the existing mSDMs that account for misclassification in occupancy models. Wright et al. (2020) provided a framework to account for the homogeneity in the classification process, and our model is connected to this by using the relationship between the multinomial and the Poisson distribution (Steel, 1953) for the observation process as well as using a species‐by‐species constant model for the classification process (Table 3). Wright et al. (2020) further provided arguments that their proposed models were generalised forms of models for the binary detection of two species (Chambert, Grant, et al., 2018), single species with count detections (Chambert et al., 2015) and single species with binary detections (Chambert, Waddle, et al., 2018). Since our proposed framework can be seen as a heterogeneous version of Wright et al. (2020), the classification component of our framework is also a generalisable form of the models in Chambert et al. (2015), Chambert, Grant, et al. (2018) and Chambert, Waddle, et al. (2018). Spiers et al. (2022) provided an individual‐level semi‐supervised approach that estimates species misclassification with occupancy dynamics and encounter rates, and our model is connected to this if we assume a homogeneous classification process (that is $\omega_{1\mathrm{jk}}=0$ for all true states *j* and reported states *k*) and assume that there is no occupancy sub‐model for the ecological process (Table 3).

**TABLE 3:** Extensions of our proposed models from homogeneous classification process studies done by Spiers et al. (2022) and Wright et al. (2020).

Author	Model framework	Link to our model
Wright et al. (2020) and the models their proposed framework generalises such as Chambert et al. (2015), Chambert, Grant, et al. (2018) and Chambert, Waddle, et al. (2018)	*Ecological process: absolute counts* $z_{\mathrm{js}}∼\text{Bernoulli}\left(\psi_{\mathrm{js}}\right)$ $\left[V_{\mathrm{jks}},|,z_{\mathrm{js}}=1\right]∼\text{Poisson}\left(\lambda_{\mathrm{jks}}\right)$	*Ecological process: relative abundance* Assume no occupancy sub‐model and for each individual, then $\left[V_{\mathrm{jks}}\right]∼\text{Categorical}\left(\lambda_{\mathrm{jks}}/\sum_{j}\lambda_{\mathrm{jks}}\right)$
	*Observation process:* $\left[Y_{\mathrm{jks}},|,V_{\mathrm{jks}}=v_{\mathrm{jks}},,z_{\mathrm{js}}=1\right]∼\text{Multinomial}\left(v_{\mathrm{jks}},|,\Omega_{\mathrm{jk}}\right)$, where $\Omega_{\mathrm{jk}}∼\text{Dirichlet}\left(\alpha \right)$	*Observation process:* $\left[Y_{\mathrm{ijs}},|,V_{\mathrm{ijs}}=v_{\mathrm{jks}}\right]∼\text{Categorical}\left(\Omega_{\mathrm{jks}}\right)$, where $\Omega_{\mathrm{jks}}$ can be chosen as any of the homogeneous models described in Table 2
Spiers et al. (2022)	*Ecological process: occupancy dynamics and encounter rates* $z_{\mathrm{jst}}∼\text{Bernoulli}\left(\psi_{\mathrm{jst}}\right)$ $\left[V_{\text{jist}}\right]∼\text{Categorical}\left(\frac{\lambda_{\text{jist}}z_{\mathrm{jst}}}{\sum_{j}\lambda_{\text{jist}}z_{\mathrm{jst}}}\right);$	*Ecological process: relative abundance* Choose t=1 and ignore the occupancy sub‐model. $\left[V_{\mathrm{jis}}\right]∼\text{Categorical}\left(\frac{\lambda_{\mathrm{jis}}}{\sum_{s}\lambda_{\mathrm{jis}}}\right);$
	*Classification process:* $\left[Y_{\mathrm{jis}},|,V_{\mathrm{jis}}\right]∼\text{Categorical}\left(\Omega_{\mathrm{jk}}\right)$, where $\Omega_{\mathrm{jk}}∼\text{Dirichlet}\left(\alpha \right)$	*Classification process:* $\left[Y_{\mathrm{jis}},|,V_{\mathrm{jis}}=1\right]∼\text{Categorical}\left(\Omega_{\mathrm{jks}}\right)$, where $\Omega_{\mathrm{jks}}$ can be chosen as any of the homogeneous models described in Table 2

*Note*: The table specifies the ecological process model for Wright et al. (absolute abundance model), Spiers et al. (occupancy dynamics and encounter rate model) and ours (relative abundance model); and also the observation model for Wright et al. and Spiers et al. (homogeneous classification process with classification probabilities simulated from Dirichlet distribution) and ours from heterogeneous models described in Table 2. Since our framework extends the work done by (Wright et al., 2020), it is safe to say that the classification component of our proposed framework are also generalised forms of Chambert et al. (2015), Chambert, Grant, et al. (2018) and Chambert, Waddle, et al. (2018). The index *j* refers to the true state identity, *k* refers to the reported state identity, *s* refers to the location in Spiers et al. (2022) and Wright et al. (2020) but refers to individuals in this study, *i* refers to the visit, and *t* refers to the year. In addition, the random variable *Y* refers to the reported observations, *V* to the verified observations and *z* to the occupancy state of the individuals.

### 2.4 Simulation study

To demonstrate how our proposed model works and its use in prediction, we performed a simulation study using *J* = 2 true states and *K* = 3 reported states over 1000 sites (we assume the locations in the simulations are discrete). We simulated two covariates for the ecological process model and one for the observation model, all from a Normal distribution with a mean of 0 and variance of 1. The ecological process intensity was simulated from Equation (5). The intercepts of the model for the two true states were chosen as $\beta_{01}=-1$, $\beta_{02}=0$ and the covariate effect for each true state was chosen as $\beta_{11}=4$, $\beta_{12}=-2$, $\beta_{21}=0$ and $\beta_{22}=0$ (that is, state 2 is used as a reference category). The intercept and covariate effect for the observation process was chosen as follows:

$$\omega_{0}\;=\left[\begin{array}{ccc} 2 & 0.5 & 0\\ 1 & 1 & 0 \end{array}\right];\omega_{1}=\left[\begin{array}{ccc} 3 & -1 & 0\\ -1 & 1 & 0 \end{array}\right].$$

These values were chosen to obtain significant sample sizes of misclassified states. We simulated 200 datasets with a heterogeneous classification process using the variable model in Table 2 (referred to as the ‘full model’), 200 with a homogeneous classification process by assuming $\omega_{1}=0$ (a matrix of zeros) in Equation (3) (referred to as ‘reduced model’) and another 200 with the covariate effect for the classification process (using the variable model in Table 2) that is correlated to the ecological process covariate (referred to as ‘correlation model’). The first simulated dataset explored modelling heterogeneity's effect on the classification process, whereas the latter explored the effect of having correlated covariates for the classification and ecological process. The second assessed the effect of overfitting the classification process model (adding heterogeneity to the classification process when it should be homogeneous). Moreover, we assessed the impact of the number of misclassified samples on the mSDMs predictive performance. We increased the principal diagonal components of $\omega_{0}$ by 6 to obtain a reduction in the number of misclassified samples simulated. The cross‐tabulation between the true state and reported state samples across all the 200 simulations is summarised in Appendix S2: Table S1. We did not explore the effect of failing to account for misclassification in this study since it has been well explored in the literature.

We randomly withheld 200 of the true state identities for each dataset simulated as our validation sample. The number of validation samples were not varied since Spiers et al. (2022) found that the number of validation samples had a modest effect on the model's predictive ability. We fitted the model under the various scenarios described in Table 2 to the data and evaluated the model's predictive performance on the validation sample.

### 2.5 Case study: Gulls dataset

The proposed model was used to analyse a gull dataset downloaded from GBIF (GBIF.Org, 2022). The database hosts over 2 billion occurrence observations with over a million observers (website visited on 17th February 2023). We were interested in the iNaturalist records since they have community verifications (Matheson, 2014). The observers collected these occurrence records and uploaded their observations with images and/or sounds that allowed for verification. The reported observations go through iNaturalist community verification and are accepted as research grade when two‐thirds of the community agreed to the taxon identification (Ueda, 2020), at which point they are published on GBIF. We assumed the community‐accepted taxon name is the true state V. We checked the iNaturalist platform to track the identification process of the observations and use the first reported identification as the reported state Y.

We obtained observations for some species of gulls in Denmark, Finland and Norway from 2015 to 2022. Specifically, we selected great black‐backed gulls (*Larus marinus*), herring (*Larus argentatus*), common gulls (*Larus canus*) and lesser black‐backed gulls (*Larus fuscus*) because the iNaturalist website reported that these species are commonly misclassified as the other. Any other species reported apart from the above‐mentioned species were labelled as ‘others’. We used annual precipitation (accessed from the raster package; Hijmans et al., 2015) as the ecological process covariate in the model since it has been noted in some literature to affect the distribution of sea birds such as gulls (Algimantas & Rasa, 2010; Jongbloed, 2016).

The data obtained were presence‐only records. Exploratory analysis revealed that there were no multiple observations at the same location for our selected species. Therefore, we assumed that our locations were discrete and treated the data as a marked process, where the individual species reported at a location is given a value of 1 and 0 for the other species in this study. If we had a species list, we could have modelled it as a repeated marked process at the same location and treated the sites as a random effect. Out of the 3737 presence‐only records retrieved, 964 were common gulls, 333 were great black‐backed gulls, 1091 were herring gulls, 339 were lesser black‐backed gulls, and 10 were others.

Citizen science data are known to be affected by several sources of bias. Some common biases are spatial bias (Johnston et al., 2022; Tang et al., 2021) and misclassifications (Johnston et al., 2022; Tulloch et al., 2013). We only accounted for the misclassifications in this study, as we are interested in explaining the classification process and not making inferences about the abundance of the gulls. Citizen scientists have been reportedly known to correctly classify species as they gain experience reporting the species (Vohland et al., 2021). We, therefore, modelled the variation in the classification process by using the number of reports made by each observer as a covariate in the classification process. If an observer has more than 10 observations, the extra number of observations was calibrated at 10. We used this number of observations for an observer as a measure of experience (Johnston et al., 2018; Kelling et al., 2015), although there are other indices for measuring effort or experience of the citizen scientist (Santos‐Fernandez & Mengersen, 2021; Vohland et al., 2021).

We also used an ML algorithm to obtain prediction scores (specifically *F*_1_ score) for our downloaded data's possible true state identity. The ML algorithm was a Convolutional Neural Network (a modified form in Koch et al., 2022) trained with data from all citizen science observations of any species in Norway. Since the ML algorithm is trained with all bird data from GBIF in Norway, we trained all our six study scenario models summarised in Table 2 with all data for our selected gull species in Norway and all data reported before 2022 in Finland and Denmark and used all data reported in 2022 in Finland and Denmark as our validation sample. The summary of the classifications (true and false positives) in the training and validation sample is presented in Appendix S2: Tables S2 and S3.

### 2.6 Fitting and evaluating the model

We ran all the analyses with the Bayesian framework using the Markov chain Monte Carlo approach in the NIMBLE package (de Valpine et al., 2017) from the R software (R Core Team, 2022). We chose the priors for all ecological process model parameters from a normal distribution with a mean of 0 and standard deviation of 10, and we chose the priors for the observation model parameters from Normal distribution with a mean of 0 and standard deviation of 1. For the scenarios: constant and main model, we chose the priors of the confusion matrix (Ω) from the Dirichlet distribution with parameter alpha (*α*), which has a prior of an exponential distribution with mean 1.

We ran 3 chains, each with 10,000 iterations; the first 5000 iterations were chosen as the burn‐in. We checked the convergence of the models by visually inspecting the trace plots and ignoring models with a Gelman‐Rubin statistic (Brooks & Gelman, 1998) value >1.1. We kept a fifth of the remaining samples in each of the chains.

We used accuracy (the proportion of predicted true state identities from all the predictions of the validation samples), precision (the proportion of mismatched true states in the validation samples that were correctly classified from the predictions) and recall (the proportion of correct true states in the validation sample retrieved from the predictions) as performance metrics. We used a Bayesian approach and got the posterior distributions for the parameters. The posterior median is estimated and higher values of the validation metrics indicated the preferred model. We also checked how well the model estimated the ecological process parameters ($\beta_{0}$, $\beta_{1}$ and $\beta_{2}$) and the classification process parameters ($\omega_{0}$ and $\omega_{1}$) by estimating the bias (difference between the true value and the estimated value) and precision of the parameters.

## 3 RESULTS

### 3.1 Simulation study

#### 3.1.1 Predictive performance

**FIGURE 1:**
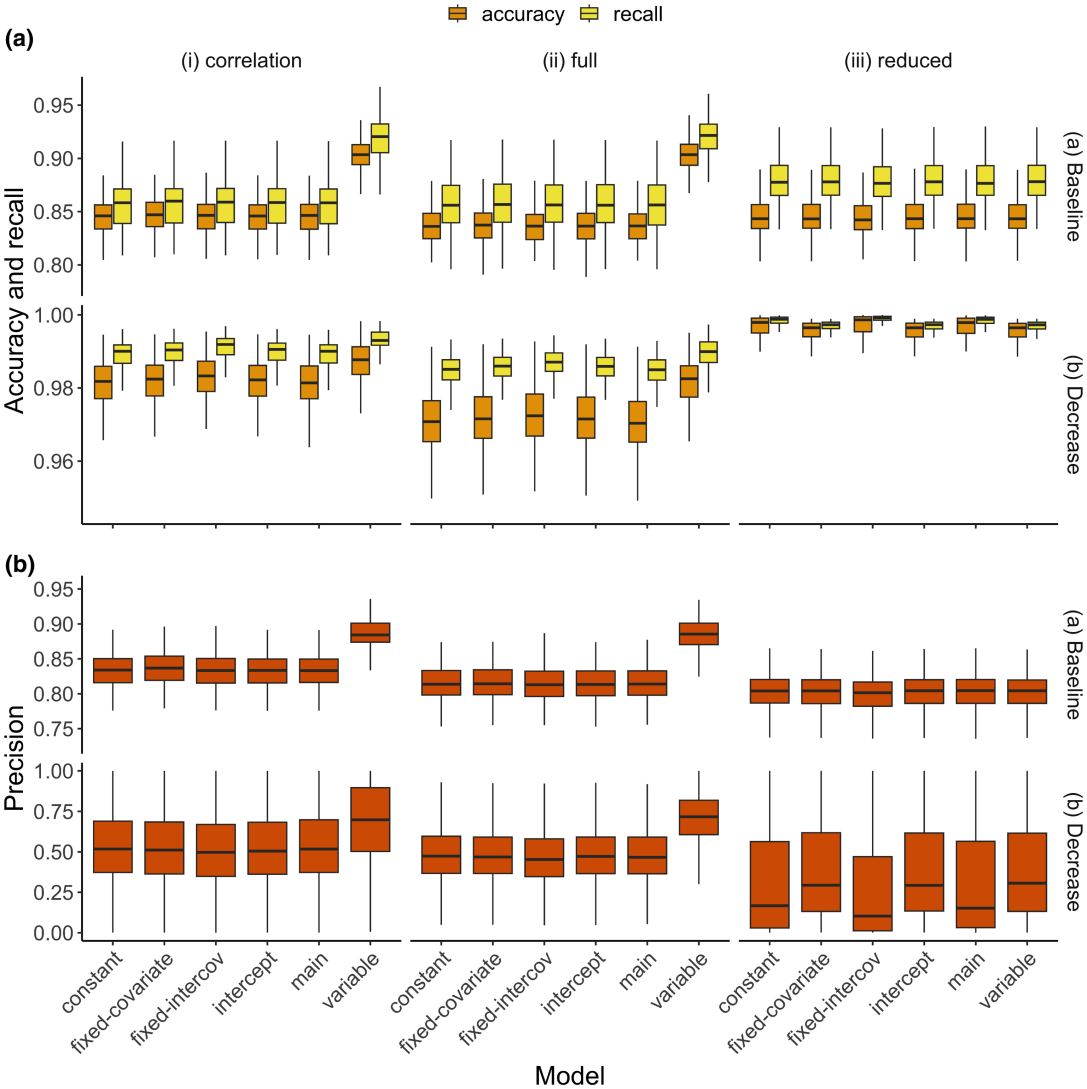
Boxplot of validation metrics (accuracy, precision, recall) from the six study models defined in Table 2 on the two hundred (200) withheld samples out of the thousand (1000) samples simulated in each dataset. Accuracy is the proportion of withheld samples that were correctly classified, recall is the proportion of correctly classified samples that were retrieved from the withheld samples, and precision is the proportion of the misclassified samples that were correctly classified. Each boxplot shows the median and the interquartile range (25–75% quartiles). Each column shows the type of model used to simulate the dataset: ‘full’ refers to using the variable/covariate model in Table 2, ‘reduced’ refers to using the intercept model in Table 2 and ‘correlation’ refers to using the variable model in Table 2, but with correlated ecological and observation process covariates. The rows correspond to changes made to the number of misclassified samples in the simulated dataset: ‘Baseline’ refers to using the values defined in Section 2.4 and ‘Decrease’ refers to reducing the number of misclassified samples by diagonal elements of ω by 6 as described in Section 2.4.

We illustrated the gain in model performance by using the accuracy, recall and precision of our model's predictions (Figure 1a,b). When data was simulated from the full and correlated models, there was a strong indication that the predictive performance of mSDMs improved when the variability in the classification process was included. That is the ‘variable’ model performed best for the full and correlation models with the highest accuracy, recall and precision values (Figure 1a(i–ii),b(i–ii)). The simplified heterogeneous models (fixed intercov and fixed covariate), however, did not perform any better than the homogeneous models (Figure 1a(i–ii),b(i–ii)). This suggested that simplifying the heterogeneous classification model did not improve predictive performance, and the heterogeneous model that captures the entire variability (in this case, variable model; Table 2) would be the best predictive model. When the classification process covariate was modelled as part of the observation process (main model), the model's predictive performance also performed similarly to the homogeneous models (Figure 1a,b).

Overfitting a homogeneous classification process with a heterogenous one did not have any effect on the mSDM's predictive performance (Figure 1a(iii),b(iii)). We expected the overfitted heterogeneous models to have poor predictive performance (Montesinos López et al., 2022), but the heterogeneous and homogeneous performed similarly (with equal recall, accuracy and precision across all six study models). The Bayesian variable selection probability indicated that the homogeneous classification model was better (with the probability of including classification covariates in heterogeneous models being 0.359 ± 0.012; Appendix S2: Table S4). Although the simplified heterogeneous models did not yield improvement in predictive performance, they performed similarly to the variable model in the variable selection process.

#### 3.1.2 Effect of number of misclassified samples

As we increased the number of misclassified samples in our simulated data, the precision increased by on average 30% and accuracy and recall increased by 6% (Figure 1a(i–ii),b(i–ii)). This decrease in accuracy and recall could be attributed to the reduced number of correct classifications in the simulated data as the number of misclassified samples increased (Appendix S2: Table S1). Moreover, the observation model parameters were estimated better when the number of misclassified samples was higher, leading to the high precision of predictions (Appendix S2: Figures S2 and S3). This suggested that our proposed model will be beneficial when one has many misclassified samples.

#### 3.1.3 Bias in observation and ecological process parameters

Although failure to account for misclassification in mSDMs can result in biased ecological process model parameters (Spiers et al., 2022; Wright et al., 2020), any method used to account for misclassification in mSDMs has a small effect on the accuracy and precision of the ecological process parameters. The bias of the ecological process parameters was consistently low for all six models, and the coverage was higher for all the scenarios under the full and reduced model (Appendix S2: Figure S1). All the scenarios studied accounted for misclassification of some sort, thereby correcting for the bias in the observation parameters estimates (Spiers et al., 2022; Wright et al., 2020). The observation model parameters were estimated more accurately for the variable model than the other models (Appendix S2: Figures S2 and S3). This was only possible in the case where we had enough misclassified samples. This suggests that if the objective of a study is to predict true state identity with mSDMs, then modelling the full heterogeneity can improve predictive performance; if the aim is inference on true state distribution, then heterogeneous models may not provide any advantage over homogeneous models.

### 3.2 Case study: Gull dataset

All six study scenario models performed equally well regarding their predictive performance with high accuracy and recall but smaller precision (Table 4). The poor precision value could not be attributed to the insignificance of the classification covariate (observer experience) in explaining the heterogeneity in the classification process since the variable selection probabilities are closer to 1 (Table 4) but to the small misclassification sample sizes (Appendix S2: Tables S2 and S3). However, the precision increased from 10% to 80% (i.e. we were able to correctly classify eight out of the ten misclassified samples) when the heterogeneity in the classification process was accounted for by using the prediction scores from the Machine learning algorithm (Table 4). The ML algorithm's prediction scores were individual observation‐specific, which provided direct information to the observation process model. However, the six classification models depended on the misclassified sample size to capture the heterogeneity in the classification process. This suggests that one remedy to improve mSDM's predictive performance for data with very small misclassified samples is to use ML weights to account for heterogeneity in mSDMs.

**TABLE 4:** Validation metrics of the models under study on the withheld gull dataset.

Method	Accuracy	Precision	Recall	Variable selection probability
Variable/Covariate	0.97	0.1	0.99	0.71
Constant	0.97	0.1	0.99	–
Intercept	0.97	0.1	0.99	–
Main	0.97	0.1	0.99	0.29
Fixed intercov	0.97	0.1	0.99	0.70
Fixed covariate	0.97	0.1	0.99	0.71
Machine Learning	0.89	0.8	0.90	–

*Note*: The accuracy is the proportion of correctly classified validated data, the precision is the proportion of mismatched identities that were correctly matched and recall is the proportion of correctly matched identities that were recovered. The number of validated samples was 384 out of which 10 were mismatched.

**FIGURE 2:**
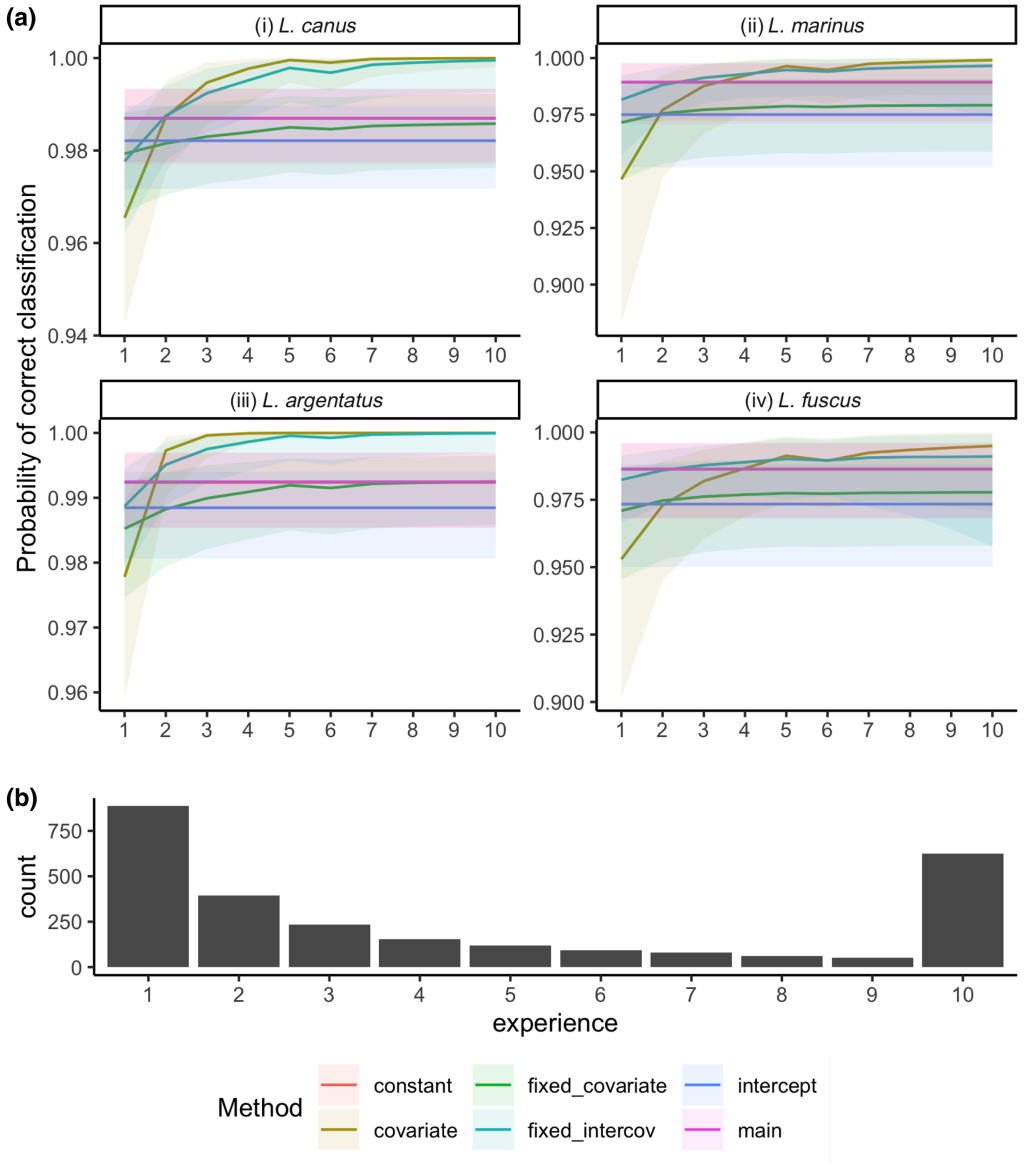
Summary of results from the model fit to gull dataset showing (a) Probability of correct classification for the common (*Larus canus*), herring (*Larus argentatus*), great black‐backed (*Larus marinus*) and lesser black‐backed gulls (*Larus fuscus*) and (b) the distribution of the experiences of the observers used in the modelling. The ribbon around the correct classification probability estimates represents the 95% credible interval of the estimates.

Although the study scenario models had smaller precision, it was observed that the probability of correctly classifying the gull species in Denmark, Finland and Norway increased with the experience of the observer (Figure 2). The pattern showed that observers have a higher chance of making mistakes on their first few reports, and they get better as the number of reports increased (Vohland et al., 2021).

## 4 DISCUSSION

The main objective of this paper was to propose a general framework to account for misclassifications from imperfect classifications (such as those from surveys) and uncertain classifications (from automated classifiers) in mSDMs. This work builds on previous work by Spiers et al. (2022); Wright et al. (2020) by accounting for the heterogeneity in the classification probabilities while allowing the classified categories to be more than the verified species (such as unknown species, morphospecies etc.). Moreover, we assessed the effect of overfitting a homogeneous classification process on the predictive performance of mSDMs and provided ways of checking the overfitting of the classification process model.

Our study bridges the knowledge gap in the literature on accounting for misclassification in mSDMs by modelling the heterogeneity in the classification process. Observation errors such as imperfect detection, sampling biases and misclassification, among many others, are inevitable in biodiversity data (Bird et al., 2014; Kéry & Royle, 2020; Miller et al., 2013). In this study, we accounted for only misclassification in the observation process. It is worth stating that the misclassification we accounted for could lead to both false positives and negatives in the biodiversity data. To model these misclassifications in this study, we presented the ecological process as one model and the observation process as another model in a hierarchical form. Under the assumption that the classification of observations is done on the individual level, we modelled the classification probabilities for each true state identity as a multinomial generalised linear model. This specification generalises the modelling of the observation process to model effects of covariates as fixed or random effects or both. For example, one can estimate the classification probabilities of each observer in volunteer‐collected data by assuming a random observer effect. This formulation for the classification process also mitigates the modelling problems of using the Dirichlet distribution as the prior for the classification probabilities (Spiers et al., 2022).

Furthermore, the specification of a separate state‐space model for the ecological process in the proposed framework allows the use of various multi‐species models (such as joint species distribution models (Ovaskainen & Abrego, 2020; Tobler et al., 2019), Royle‐Nichols model for abundance (Royle & Nichols, 2003), among many others) to model the distribution of the true states. With the classifications assumed to be done on individual sample levels, an ecological process model can be defined to link the true and reported states appropriately. For example, if species list (obtained from checklists as provided by eBird; Sullivan et al., 2014; Johnston et al., 2021) are used to model species distributions, the record at each location can be treated as repeated observations (where each observation refers to a different individual species) at the same location. Our simulation study showed that the proposed model framework could estimate the process model parameters (with the bias of estimated parameters close to zero; Appendix S2: Figure S1), an observation noted in previous studies that use observation confirmation design to model misclassification in mSDMs (Kéry & Royle, 2020; Spiers et al., 2022; Wright et al., 2020). We have shown that the ecological process model presented in this study is a simplified form of occupancy and abundance models (in the sense that it ignores species occurrence) that account for misclassification (Table 3), so we believe our proposed framework can be extended to any design used to collect and verify data on the true states (for example, point processes, distance sampling, site confirmation and other multi‐method design, etc.), and any model used to fit the data (for example, multi‐state occupancy model (Kéry & Royle, 2020), joint species distribution models (Ovaskainen & Abrego, 2020; Tobler et al., 2019)). Although such extensions are possible, significant computational and/or practical challenges must be explored in future work. For example, joint species distribution models would estimate residual correlations between species in the ecological process model while simultaneously estimating misclassification probabilities among the species in the observation model. These additional parameters can cause the models to be non‐identifiable or computationally expensive.

Modelling the ecological process with more complex models than the relative abundance models used in the study would add another level of hierarchical structure to the proposed framework (for example, modelling detection probability or true occupancy state). This complexity could introduce confounding of the ecological and observation process model parameters and, with frequentist estimation approaches, make the likelihood multi‐modal (Kéry & Royle, 2020). This study did not explore such issues; further work can be done on this. A possible solution in the Bayesian framework to avoid such confounding issues would be to model the different processes with separate covariates, choose a good prior for the mSDM parameters and use repeated survey visit data to model the observation process (Kéry & Royle, 2020). Moreover, the identifiability or confounding issues could be tackled by using data with much information on detection and false positive detections, such as those derived from acoustic surveys (Clement et al., 2022) and integrating occupancy or count data that are not susceptible to misclassification, such as those from camera traps to those with misclassifications (Doser et al., 2021; Kéry & Royle, 2020).

Accounting for the heterogeneity in the classification process increases the predictive performance of mSDMs. The homogeneous classification models may sometimes be unable to explain the variation in the observation process (Conn et al., 2013), leading to poor model predictive performance due to overfitting (Montesinos López et al., 2022). The simulation study showed a 30% increase in precision and a 6% increase in accuracy and recall when the heterogeneity in the classification process was accounted for in the mSDMs (Figure 1a,b). However, there was no change in predictive performance when a heterogeneous classification model overfitted a homogeneous classification process (Figure 1a,b) due to the small classification covariate effect size, observed from the bias of parameter estimates and low Bayesian variable selection probability (Appendix S2: Figures S1–S3). Since the predicted posterior probability for the true state's identity heavily relies on the weights from the misclassification probability (Appendix S1), failure to account for heterogeneity in the classification process would mean our posterior probability would be incorrectly estimated. The incorrectly predicted probability would lead to the underestimation of the prediction of the ranges of coverage and possibly abundance in the true states (Molinari‐Jobin et al., 2012). It must be noted that this study did not compare mSDMs that account for misclassification to those that do not account for misclassification but can further infer from previous studies that perform this comparison that failure to account for any misclassification would also lead to underestimation of prediction ranges and species distribution (Clare et al., 2021; Ferguson et al., 2015; Miller et al., 2015; Wright et al., 2020).

Fitting a more complex ecological process model with the covariate that explains the heterogeneity of the classification process does not provide enough information to improve the mSDM's predictive performance. Previous studies have shown that the estimates of the ecological process model inform the estimation of the classification probabilities (Spiers et al., 2022), but the variability in the classification process cannot be inferred from variability in the ecological process model (Figure 1a(i–iii), Appendix S1). Ecologists should, therefore, model the variability in the classification in its process model to gain the advantage in the mSDMs predictive performance.

Our model was parameterised with volunteer‐collected gull data. These volunteer‐collected data have several sources of bias in their generation, such as spatial bias, and misidentification of species, among many others. We acknowledge that all these sources of biases may be present in the data, but we only modelled the misidentification of species by using the number of previously collected data as a proxy measure for the observer's experience in the classification process model. The predictive performance of the homogeneous and heterogeneous models was approximately the same due to small misclassified samples (19 misclassified out of 1382 samples in training data (Appendix S2: Table S2)) and 10 misclassified out of 378 samples in validation data (Appendix S2: Table S3). However, the estimated covariate effect shows how the experience affects the probability of classifying a new observation. Specifically, the probability of correctly identifying the correct species increases with the observer's experience, as is noted in some literature (Johnston et al., 2018; Kelling et al., 2015; Santos‐Fernandez & Mengersen, 2021; Vohland et al., 2021). Therefore, there is a trade‐off between the model's ability to correctly classify mismatched data (precision) and understanding the covariate's effect driving the classification process when there are relatively small misclassified samples.

The inclusion of ML prediction scores in the mSDMs to account for the heterogeneity in the classification process increased the precision of our predictions by 70% (Table 4). These ML prediction scores are observation‐specific and provide much information about the classification process to increase the precision of the model. The information from the ML does not depend on the misclassified sample sizes but on the quality of the images (Koch et al., 2022), making them advantageous to use in accounting for heterogeneity in the classification process when misclassified sample sizes are small (like we have in our gull data).

This study leaves room for further work to be done. We used 1000 locations in our simulation study and 2737 locations in the case study. In some real‐world applications, such as those that use acoustic survey data, collecting data at a few sites is feasible due to how expensive it is to collect the data (Darras et al., 2018; Doser et al., 2021; Efford et al., 2009). Further studies can explore the impact of the number of study sites on the performance of the proposed framework. Moreover, this study used two true states and three reported states, and the case study used four true states and five reported states. Increasing the number of true states and reported states may affect the performance of our proposed model, which we have left for further studies.

The proposed model framework in this study is flexible and can be generalised into any species distribution model and integrated distribution model. The framework proposed fits into the frameworks provided by Spiers et al. (2022) and Wright et al. (2020) and any framework their study generalises. Our proposed classification process model, MMGLM, improved the predictive performance of mSDMs, but it heavily relies on the misclassified sample size. Furthermore, the confusion matrix defined in the model framework allows for the classification of different taxonomic groups, as opposed to just the species‐by‐species confusion matrix in Wright et al. (2020) and including morphospecies in the classification categories (Spiers et al., 2022). This will make it possible for citizen science data analysts to account for the misclassification of data at any level in the data collection process. We recommend that variable or model selection is performed during the analysis to check for overfitting. Moreover, ecologists should explore using ML prediction scores (where the prediction scores are available) as weights in mSDMs that aim at predicting true state distributions, especially when the data has a small misclassified sample size.

## AUTHOR CONTRIBUTIONS

**Kwaku Peprah Adjei:** Conceptualization (equal); formal analysis (lead); methodology (equal); writing – original draft (lead); writing – review and editing (lead). **Anders Gravbrøt Finstad:** Conceptualization (supporting); funding acquisition (equal); supervision (supporting). **Wouter Koch:** Formal analysis (supporting); methodology (supporting); writing – original draft (supporting); writing – review and editing (supporting). **Robert Brian O'Hara:** Conceptualization (equal); funding acquisition (lead); methodology (equal); writing – original draft (supporting); writing – review and editing (equal).

## CONFLICT OF INTEREST STATEMENT

The authors declare no conflict of interest.

## Supporting information


Appendix S1.

Appendix S2.

## Data Availability

The Gulls data used for this paper can be downloaded from GBIF (https://doi.org/10.15468/dl.h24bp5). All codes used for this paper are available at data dyrad with https://doi.org/10.5061/dryad.0rxwdbs51.
