# Supplementary material for: Modelling heterogeneity in the classification process in multi‐species distribution models can improve predictive performance
Source: Ecol Evol. 2024 Mar 7;14(3):e11092. doi: 10.1002/ece3.11092 (PMC10918728; doi:10.1002/ece3.11092)
Supplement: Supplementary file 1 — Appendix S1. [file ECE3-14-e11092-s001.pdf]

Supplementary Information One (S1) for 'Modelling heterogeneity in classification process in multi-species distribution models improves predictive performance.'

Kwaku Peprah Adjei[1,2, 5], Anders Finstad[2,3], Wouter Koch[2,4], Robert B. O'Hara[1,2]

[1]Department of Mathematical Sciences, Norwegian University of Science and Technology, Norway

[2]Center for Biodiversity Dynamics, Norwegian University of Science and Technology, Norway

[3]Department of Natural History, Norwegian University of Science and Technology, Norway

[4]Norwegian Biodiversity Information Center, Trondheim, Norway

[4]Norwegian Institute for Nature Research, Trondheim, Norway

Representation of Equation (2) and (3).

We want to show the relationship between the linear predictor and the classification probabilities defined in equations (2) and (3) in the multinomial logit model. Given the data with $c = 1, 2, \ldots, C + 1$ categories, Fahrmeir et al. (2013) defined the multinomial logit model as described below

$$log(\frac{\pi_c}{\pi_{C+1}}) = X'\beta, \tag{1}$$

where π_c is the probability of observing category c, π_{C+1} is the probability of observing reference category $C + 1$, β is a vector of coefficients and X is the design matrix.

Let us define k' as the reference reported state, for $k' \in \{1, 2, \ldots, K\}$. Given the definition of the linear predictor in equation (2) in the main paper, then the probability of classifying true state j as k with reference

to reported state K is:

$$\begin{aligned}
\frac{\Omega_{jks}}{\Omega_{jKs}} &= \frac{\exp\left(\zeta_{jks}\right)}{\exp\left(\zeta_{jKs}\right)} \\
&= \frac{\exp\left(\omega_{0jk} + \sum_{p=1}^{n} z_{ps}\omega_{pjk}\right)}{\exp\left(\omega_{0jK} + \sum_{p=1}^{n} z_{ps}\omega_{pjK}\right)} \\
&= \exp\left(\omega_{0jk} - \omega_{0jK} + \sum_{p=1}^{n} z_{ps}(\omega_{pjk} - \omega_{pjK})\right) \\
\implies \ln\left(\frac{\Omega_{jks}}{\Omega_{jKs}}\right) &= (\omega_{0jk} - \omega_{0jK}) + (\omega_{1jk} - \omega_{1jK}) * z_{1s} + \ldots + (\omega_{njk} - \omega_{njK}) * z_{ns};
\end{aligned} \tag{2}$$

which is the same as the multinomial logit model defined in equation (1).

References

Fahrmeir, L., Kneib, T., Lang, S. and Marx, B. (2013) *Categorical Regression Models*, 325–347. Berlin, Heidelberg: Springer Berlin Heidelberg. URL: https://doi.org/10.1007/978-3-642-34333-9_6.
