# Supplementary material for: Modelling heterogeneity in the classification process in multi‐species distribution models can improve predictive performance
Source: Ecol Evol. 2024 Mar 7;14(3):e11092. doi: 10.1002/ece3.11092 (PMC10918728; doi:10.1002/ece3.11092)
Supplement: Supplementary file 2 — Appendix S2. [file ECE3-14-e11092-s002.pdf]

Supplementary Information Two (S2) for 'Modelling heterogeneity in the classification process in multi-species distribution models can improve predictive performance.'

Kwaku Peprah Adjei[1,2,5], Anders Finstad[2,3], Wouter Koch[1,4], Robert B. OHara[1,2]

[1]Department of Mathematical Sciences, Norwegian University of Science and Technology, Trondheim, Norway

[3]Department of Natural History, Norwegian University of Science and Technology, Trondheim, Norway

[2]Center for Biodiversity Dynamics, Norwegian University of Science and Technology, Trondheim, Norway

[4]Norwegian Biodiversity Information Center, Trondheim, Norway

[5]Norwegian Institute for Nature Research, Trondheim, Norway

Table 1: Summary of sample sizes for cross-tabulation between each true-state and reported state across the 200 simulations (with standard deviation in paranthesis) for each of the three simulation scenarios (correlation, full and reduced model) described in section 2.4 in the main paper. The simulation study used two true-states (represented by the first value before the comma in the header of columns three to eight) and three reported states (represented by the second value after the comma in the header of columns three to eight). The number of misclassified samples were decreased by adding a factor of 6 to ω_{0jk} for all $j = k$.

Model	Factor	1,1	2,1	1,2	2,2	1,3	2,3
correlation model	0	226 (8.93)	236 (11.34)	72 (7.47)	184 (9.91)	24 (5.04)	57 (6.56)
correlation model	6	319.5 (9.78)	9 (2.66)	4 (1.9)	465 (176.31)	1 (177.32)	1 (0.89)
full model	0	209 (8.82)	200.5 (9.89)	104 (6.89)	205 (10.61)	28 (5.04)	53 (6.87)
full model	6	325 (9.86)	5.5 (2.33)	14 (3.19)	453 (100.58)	1 (102.24)	0 (0.79)
reduced model	0	249 (10.75)	194 (10.2)	55 (6.95)	195 (11.35)	34 (5.59)	72 (7.13)
reduced model	6	338 (9.16)	1 (1.19)	0 (0.49)	1 (225.79)	450.5 (227.63)	1 (0.65)

Table 2: Summary of classifications of the gull species - common (*Larus canus*), herring (*Larus argentatus*), great black-backed (*Larus marinus*) and lesser black-backed (*Larus fuscus*) - in the training dataset.

True-state	common	great black-backed	herring	lesser black-backed	other
common	775	0	7	0	2
great black-backed	0	254	1	1	0
herring	0	1	846	0	4
lesser black-backed	0	2	1	282	0

Table 3: Summary of classifications of the gull species - common (*Larus canus*), herring (*Larus argentatus*), great black-backed (*Larus marinus*) and lesser black-backed (*Larus fuscus*) - in the validation dataset.

True-state	common	great black-backed	herring	lesser black-backed	other
common	139	0	4	0	2
great black-backed	0	43	0	2	0
herring	0	0	161	0	1
lesser black-backed	0	1	0	34	0

Table 4: Summary of variable selection probability from the simulation studies (with standard errors in parenthesis). The first column refers to the simulation method (full, reduced and correlation), the second column indicated whether we decreased the number of misclassified samples (add 6 to the ω_{0jk} for all $j = k$ and baseline refers to using the original true model parameter values) and the next six columns refer to the study scenario type used in this study.

simMethod	trueMisclassIncrease	constant	fixed-covariate	fixed-intercov	intercept	main	variable
correlation	Baseline	0.4999(0.0023)	0.6606(0.0193)	0.6638(0.0112)	0.4998(0.0023)	0.5002(0.0025)	0.6666(0.0045)
correlation	Decreased	0.4997(0.0023)	0.4313(0.0505)	0.484(0.0634)	0.4998(0.0023)	0.4996(0.0023)	0.6663(0.0051)
full	Baseline	0.5001(0.0025)	0.6172(0.0665)	0.5872(0.0807)	0.4997(0.0023)	0.5(0.0024)	0.6666(0.0045)
full	Decreased	0.4999(0.0024)	0.4285(0.0623)	0.5125(0.0857)	0.4996(0.0023)	0.4997(0.0024)	0.6671(0.0048)
reduced	Baseline	0.5002(0.0022)	0.3539(0.0272)	0.3529(0.0233)	0.5001(0.0024)	0.5002(0.0024)	0.3357(0.0083)
reduced	Decreased	0.5001(0.0022)	0.3879(0.0081)	0.4765(0.0236)	0.4996(0.0024)	0.5001(0.0024)	0.359(0.0124)

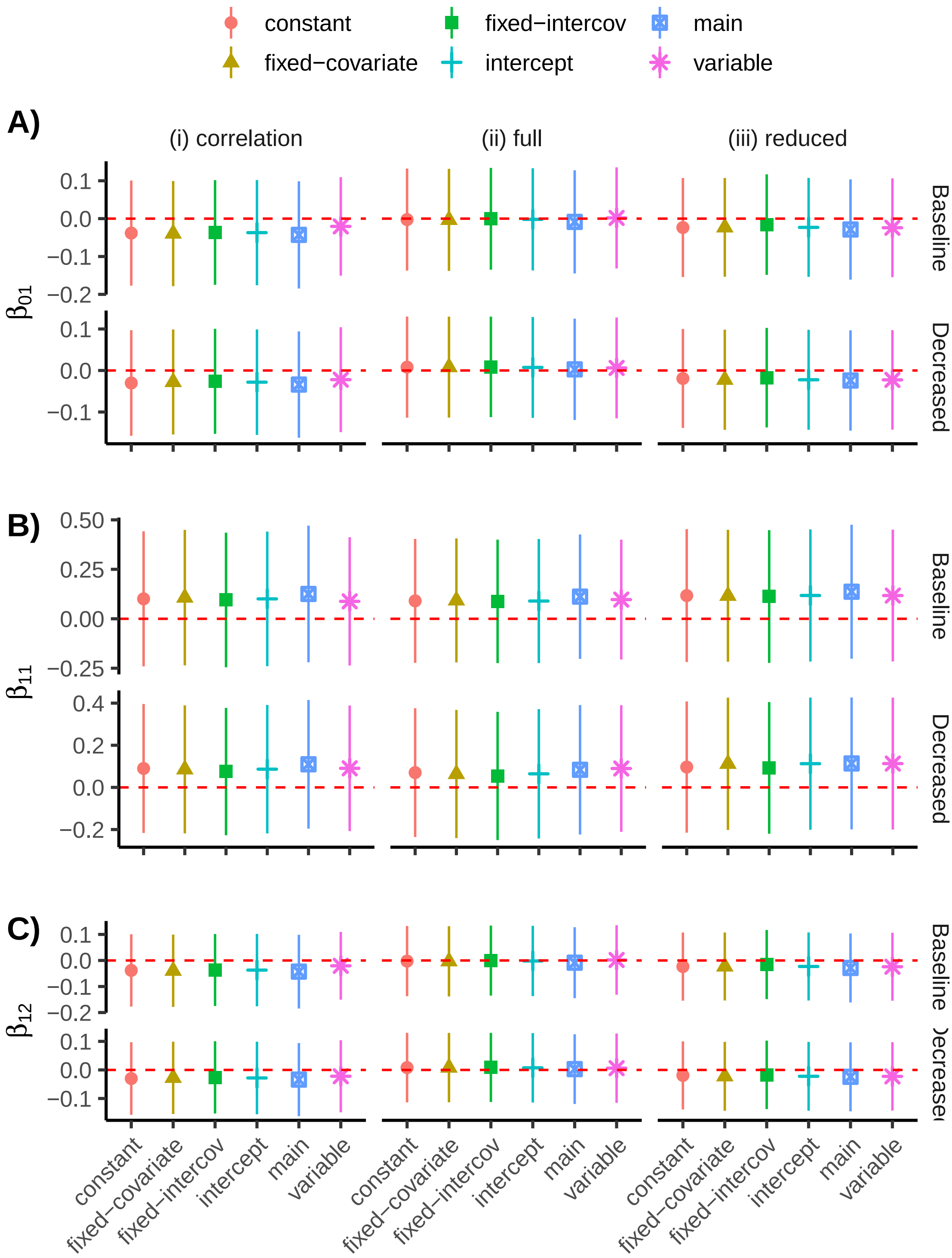

Figure 1: Error bar of mean bias in ecological process paramaters from the simulation studies (the width of the bars shows the standard devation of the bias). Each column shows the type of model used to simulate the dataset. “full” refers to using the variable/covariate model, “reduced” refers to using the intercept model and “correlation” refers to using the variable model, but with correlated ecological and observation process covariates (all three models are described in Table 2 in the main paper). The rows correspond to changes made to the number of misclassified samples in the simulated dataset. “Baseline” refers to using the values defined in section 2.4 and “Decrease” refers to reducing the number of misclassified samples by diagonal elements of by 6 as described in section 2.4 in the main paper. The red dahsed line where the bias of the parameter is 0.

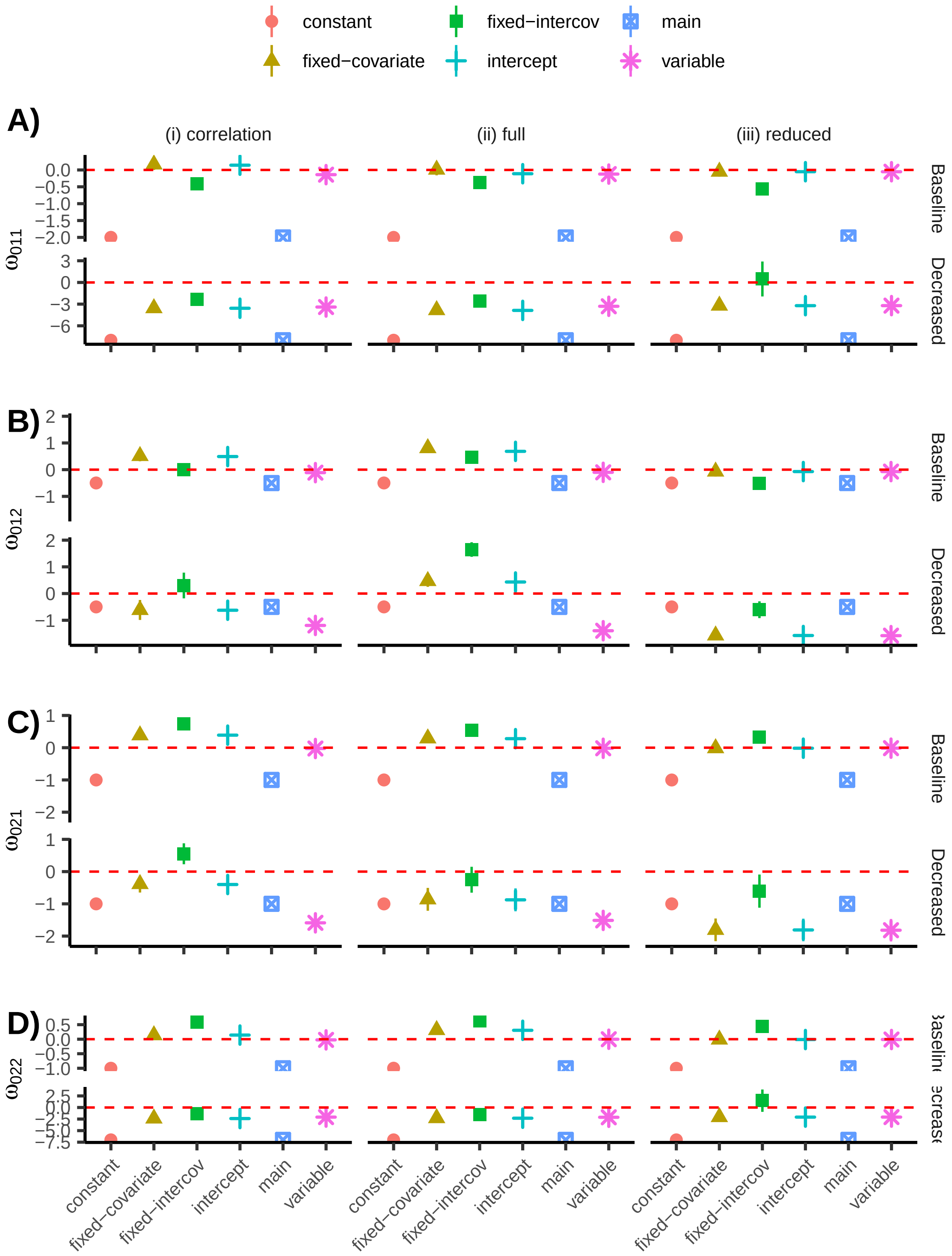

Figure 2: Error bar of mean bias in the intercept of the classification process paramaters ω_0 from the simulation studies (the width of the bars shows the standard devation of the bias). Each column shows the type of model used to simulate the dataset. “full” refers to using the variable/covariate model, “reduced” refers to using the intercept model and “correlation” refers to using the variable model, but with correlated ecological and observation process covariates (all three models are described in Table 2 in the main paper). The rows correspond to changes made to the number of misclassified samples in the simulated dataset. “Baseline” refers to using the values defined in section 2.4 and “Decrease” refers to reducing the number of misclassified samples by diagonal elements of by 6 as described in section 2.4 in the main paper. The red dahsed line where the bias of the parameter is 0.

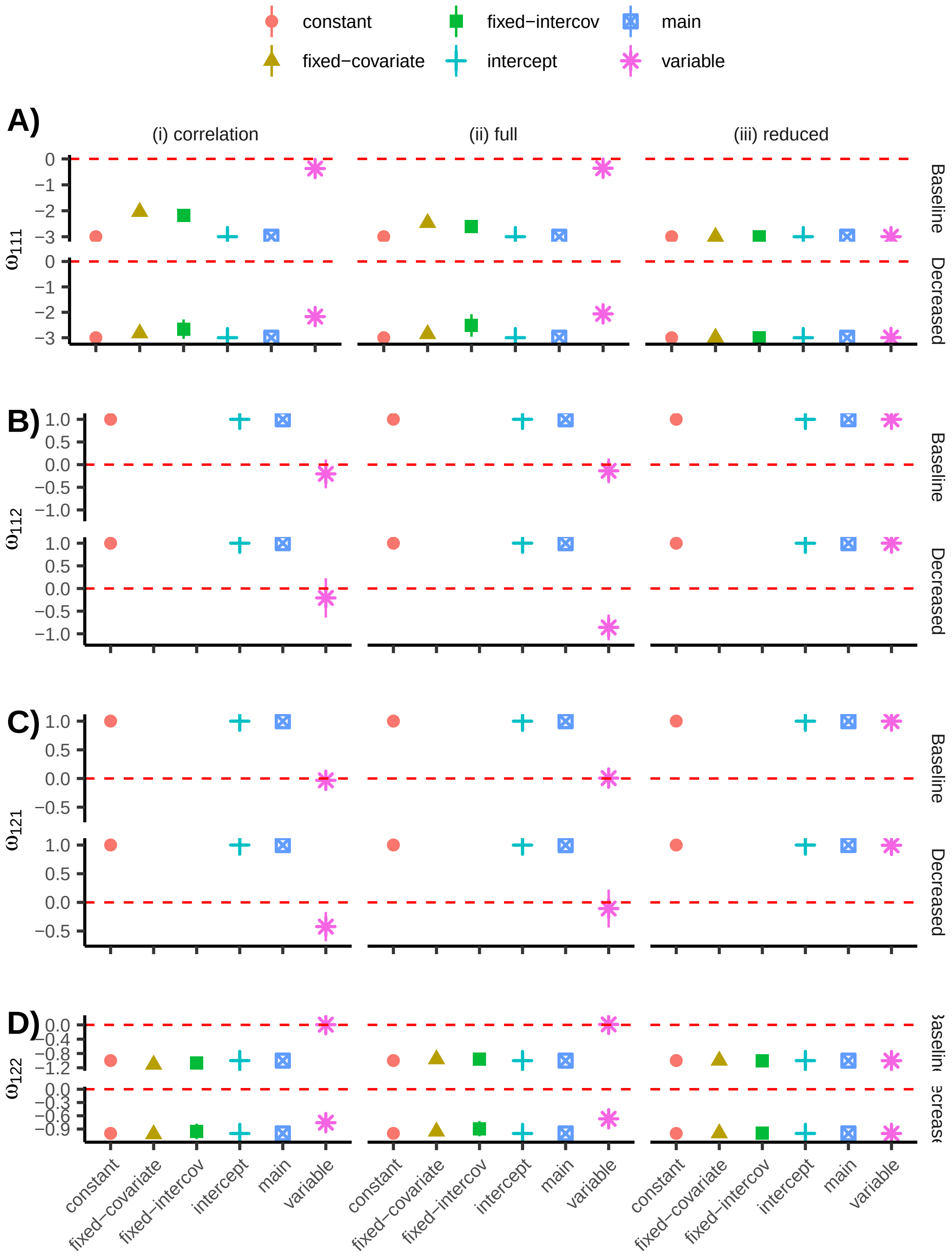

Figure 3: Error bar of mean bias in the covariate effect of the classification process paramaters ω_1 from the simulation studies (the width of the bars shows the standard devation of the bias). Each column shows the type of model used to simulate the dataset. “full” refers to using the variable/covariate model, “reduced” refers to using the intercept model and “correlation” refers to using the variable model, but with correlated ecological and observation process covariates (all three models are described in Table 2 in the main paper). The rows correspond to changes made to the number of misclassified samples in the simulated dataset. “Baseline” refers to using the values defined in section 2.4 and “Decrease” refers to reducing the number of misclassified samples by diagonal elements of by 6 as described in section 2.4 in the main paper. The red dahsed line where the bias of the parameter is 0.
